# Concomitant transcatheter occlusion versus thoracoscopic surgical clipping for left atrial appendage in patients undergoing ablation for atrial fibrillation: A meta-analysis

**DOI:** 10.3389/fcvm.2022.970847

**Published:** 2022-09-06

**Authors:** Shijie Zhang, Yuqi Cui, Jinzhang Li, Hongbo Tian, Yan Yun, Xiaoming Zhou, Hui Fang, Haizhou Zhang, Chengwei Zou, Xiaochun Ma

**Affiliations:** ^1^Department of Cardiovascular Surgery, Shandong Provincial Hospital, Shandong University, Jinan, Shandong, China; ^2^Center for Precision Medicine and Division of Cardiovascular Medicine, University of Missouri School of Medicine, Columbia, MO, United States; ^3^Department of Cardiology, Shandong Provincial Hospital, Shandong First Medical University, Jinan, Shandong, China; ^4^Department of Cardiac Surgery, Beijing Anzhen Hospital, Capital Medical University, Beijing, China; ^5^Department of Radiology, Qilu Hospital of Shandong University, Jinan, Shandong, China; ^6^Department of Endocrinology, Shandong Provincial Hospital, Shandong First Medical University, Jinan, Shandong, China; ^7^Department of Cardiovascular Surgery, Shandong Provincial Hospital, Shandong First Medical University, Jinan, Shandong, China; ^8^School of Chemistry and Chemical Engineering, University of Jinan, Jinan, Shandong, China

**Keywords:** atrial fibrillation, left atrial appendage occlusion, left atrial appendage clipping, stroke, thoracoscopy, meta-analysis, catheter ablation

## Abstract

**Background:**

Both catheter left atrial appendage occlusion combined with ablation (COA) and thoracoscopic surgical left atrial appendage clipping combined with ablation (TCA) have shown favorable outcomes in management of patients with atrial fibrillation (AFib). However, studies comparing the endpoints of both techniques are still lacking. Herein, a meta-analysis of safety and efficacy outcomes of COA versus TCA was performed in patients with AFib.

**Methods:**

Pubmed, Embase, Cochrane, and Web of Science databases were searched for retrieving potential publications. The primary outcome was the incidence of stroke during follow-up period of at least 12 months. Secondary outcomes were acute success rate of complete left atrial appendage (LAA) closure by COA or TCA, postprocedural mortality and complications, and all-cause mortality during follow-up period of at least 12 months.

**Results:**

19 studies of COA containing 1,504 patients and 6 studies of TCA with 454 patients were eligible for analysis. No significant difference in stroke and all-cause mortality was found in patients undergoing COA versus TCA after at least a 12-month follow-up (stroke: *p* = 0.504; all-cause mortality: *p* = 0.611). COA group had a higher acute success rate compared with TCA group (*p* = 0.001). COA placed the patients at a higher risk of hemorrhage during the postprocedural period compared with TCA (*p* = 0.023). A similar risk of other postprocedural complications (stroke/transient ischemic attack and pericardial effusion) and mortality was found in the COA group in comparison with TCA group (*p*>0.05).

**Conclusion:**

This meta-analysis showed that COA and TCA did not differ in stroke prevention and all-cause mortality in patients with AFib after a follow-up of at least 12 months. Postprocedural complications and mortality were almost comparable between the two groups. In the near future, high-quality randomized controlled trials exploring the optimal surgical strategies for AFib and endpoints of different procedures are warranted.

**Systematic review registration:**

[https://www.crd.york.ac.uk/PROSPERO/], identifier [CRD42022325497].

## Introduction

Atrial fibrillation (AFib), the most common sustained cardiac arrhythmia in clinical practice, affects around 2∼4% adults worldwide (1, 2). The prevalence increases markedly with aging (3) and is expected to burgeon in the next few decades (4, 5). Serious complications of AFib include thromboembolism, congestive cardiac failure, and predispose a poorer survival (6). In management of patients with AFib, the major issues are related to the arrhythmia itself and the prevention of thromboembolism.

Antiarrhythmic drugs (AADs) impose a fundamental role in rate- and rhythm-control in patients with AFib. Catheter ablation (CA) or surgical ablation (SA) has been greatly developed for refractory AFib (7–9) and hybrid ablation has shown more favorable outcomes (10). Long-term anticoagulation is recommended as the gold standard of thromboembolism prevention with oral anticoagulation (OAC) therapy.

Left atrial appendage (LAA) is the source of thrombi in > 90% of patients with AFib (11), which provides the rationale for ligation, amputation, or occlusion of LAA. In recent years, various studies have reported that both catheter left atrial appendage occlusion combined with ablation (COA) and thoracoscopic surgical left atrial appendage clipping combined with ablation (TCA) are feasible and safe. Although the outcomes of COA and TCA have been well documented separately, the outcomes of both procedures have not been compared before. Therefore, a meta-analysis was performed to compare the safety and efficacy outcomes of COA versus TCA in patients with AFib after at least 12 months of follow-up.

## Methods

This study’s protocol is registered on PROSPERO (CRD42022325497) and reported in accordance with the Meta-analysis of Observational Studies in Epidemiology guidelines (MOOSE) (12) (Supplementary Table 1). This study was conducted under the supervision of the Ethics Committee of Shandong Provincial Hospital.

### Eligibility and exclusion criteria

Eligible studies satisfied the following criteria:

(A) studies published as full-text, peer-reviewed articles, with no language restriction;
(B) recruiting adult patients;
(C) patients undergoing catheter left atrial appendage occlusion combined with ablation (COA) or thoracoscopic surgical left atrial appendage clipping combined with ablation (TCA; including hybrid ablation) due to isolated AFib;
(D) reporting the outcomes in preventing stroke in AFib;
(E) interested outcomes including stroke and all-cause mortality during follow-up as well as complications and mortality occurring in the postprocedural period;
(F) study-level data available for statistical analysis.

Exclusion criteria were:

(a) case reports, abstracts, as well as editorial comments and review articles;
(b) studies unpublished or with insufficient data;
(c) follow-up less than 12 months;
(d) surgical procedures concomitant with other intra-cardiac interventions, such as mitral valve or coronary artery bypass surgery.

In case there was overlap in the patient populations in different studies from the same center, we included only the study with the longest follow-up or largest patient cohort.

### Search strategy and data extraction

Two authors (Shijie Zhang, Yuqi Cui) searched the Pubmed, Embase, Cochrane, and Web of Science databases from 2012 to 2022 independently, for retrieval of eligible studies reported on the LAA closure in patients with AFib. For the COA group, the search terms used included: “atrial fibrillation,” “occlusion,” “closure,” “left atrial appendage,” “catheter,” “transcatheter,” “percutaneous.” For the TCA group, the search terms used were: “atrial fibrillation,” “exclusion,” “clipping,” “clip,” “occlusion,” “closure,” “left atrial appendage,” “thoracoscopic,” “minimally invasive,” “epicardial.” No search software was used. Authors were not contacted for studies that did not fulfill inclusion criteria or if data were unclear. All identified studies were screened based on their titles and abstracts by these two authors. Next, the full-text articles potentially included in this meta-analysis were re-examined to finally determine the inclusion. The reference lists were also manually checked from retrieved articles for including potential publications. Disagreements were resolved by a further discussion among all authors.

Data extraction was carried out separately by two authors (Xiaochun Ma, Shijie Zhang). Extracted data mainly included the study design and quality, demographics, baseline characteristics, and outcomes of interest. The primary outcome was defined as the incidence of stroke during a follow-up period of at least 12 months. Secondary outcomes were all-cause mortality during follow-up period of at least 12 months, acute success rate, and postprocedural mortality and complications including stroke/transient ischemic attack (TIA), hemorrhage, and pericardial effusion. The acute success of the procedure was defined as successful device implantation with no or minimal residual flow (flow ≤ 5 mm) after ablation was finished. The duration of follow-up was extracted as mean or median length. The patient-year was extracted to calculate the incidence. If reported, the patient-year was retrieved from original articles; if not, was estimated by multiplying the subject number with mean or median follow-up time. Also, disagreements were resolved by a further discussion among all authors.

### Quality assessment

The quality of all included studies was assessed by two authors independently (Hongbo Tian, Jinzhang Li). In all observational studies and non-randomized clinical trials, the risk of bias was assessed with the use of Methodological Index for Non-Randomized Studies (MINORS) score (13). In articles reporting randomized controlled trials (RCTs), the risk of bias was assessed using the RoB2 (Revised Cochrane risk of bias tool for randomized trials) (14).

### Statistical analysis

All statistical analyses were performed using the software Open Meta-Analyst for Windows 8 (2015). Study characteristics were presented as raw values and percentages for categorical variables, and as mean and 95% confidence interval (CI) for continuous variables. The *p*-values of continuous variables were computed using the metric “TX Mean” for the weighted mean difference, whereas “Untransformed Proportions” were used for calculating the *p*-values of categorical data. Due to the low complication rates and high success rate, primary and secondary outcomes were analyzed using the metric “Freeman–Tukey Double Arcsine Proportion” (15). Follow-up outcomes were measured by calculating the event number per 100 patient-years and the incidences of postprocedural complications were defined as event number divided by subject number. To compare the outcomes between two groups, the study type was used as a covariate factor for running all meta regressions (16). All statistical values were computed with 95% confidence intervals (CIs) in a binary random-effects model, and the *p*-values less than 0.05 was considered statistically significant for all estimates. For the Chi-square test of heterogeneity, a *p*-value less than 0.10 was considered statistically significant. Besides, *I*^2^ metric estimates the percentage of total variation between studies, and *I*^2^ more than 50% indicates significant heterogeneity.

## Results

### Study selection and inclusion

**FIGURE 1:**
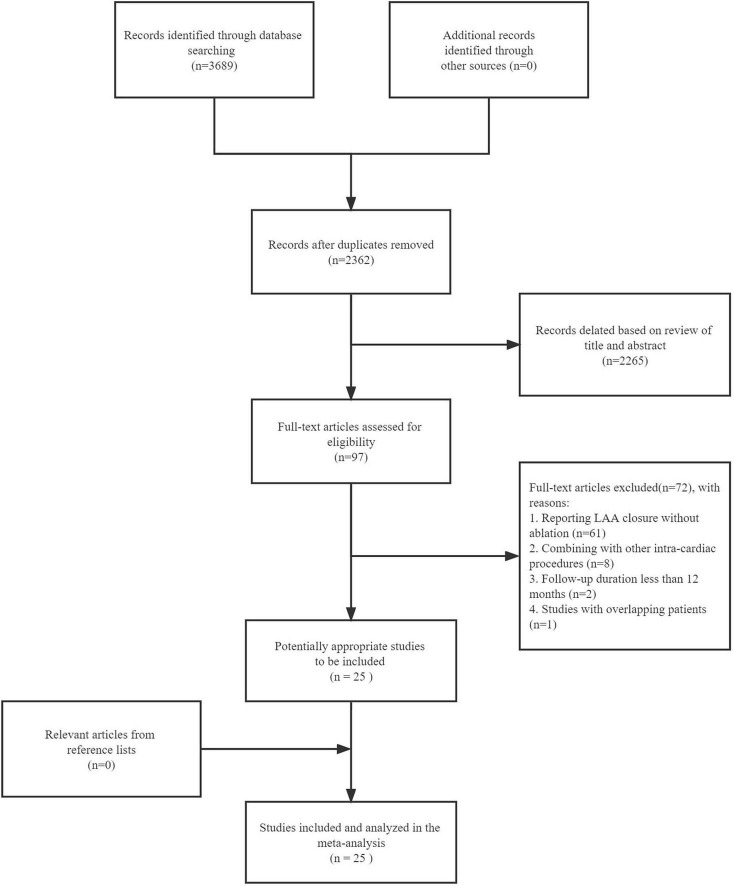
Flow-chart of systematic literature search and study inclusion.

After a digital literature search, a total of 2,362 articles were retrieved from the search after duplicates were discarded. The titles and abstracts of these studies were carefully examined and 2,265 studies were categorized as irrelevant, leaving 97 studies pending re-examination. After retrieval and check of full texts, 72 studies were omitted for not satisfying the eligibility criteria (61 for reporting LAA closure without ablation; 8 for combining with other intra-cardiac procedures; 2 for follow-up duration less than 12 months; 1 study for reporting duplicate data). Two studies (17, 18) were not excluded because their follow-up time was very close to 12 months. A consensus was reached among all authors regarding the final inclusion of studies. Figure 1 presents the flow diagram of study selection and inclusion.

### Study characteristics and quality assessment

Ultimately, 19 studies of COA containing 1,504 patients and 6 studies of TCA with 454 patients satisfied our eligibility criteria and were included in this meta-analysis. Characteristics of included studies were detailed in Table 1 (17–41). For all included studies, thrombus exclusion in the left atrium or LAA and measurements of LAA was conducted by transesophageal echocardiography (TEE) or angiography before procedures. Pulmonary vein isolation was performed which represents the cornerstone of AFib ablation and successful LAA closure was confirmed by TEE. The detailed ablation strategies of both approaches were shown in Supplementary Table 2. All included patients were administrated with a short-term OAC therapy. In the COA group, patients were prescribed an oral anticoagulant for 45 days or 3 months, followed by a single or dual antiplatelet strategy.

**TABLE 1:** Study characteristics.

Source	Design	Approach	Patients (*n*)	Male *n* (%)	Age, y (Mean ± SD)	Percentage of persAFib and (or) LSPAFib *n* (%)	Use of OAC before admission *n* (%)	CHADS2-VASc score	Follow-up (months)	Total patient-years of follow-up
Walker et al. (19)	Single-center, prospective	COA	26	20 (76.9)	63 ± 7	12 (46.2)	NA	2.6 ± 0.8	12	26
Swaans et al. (20)	Single-center, prospective	COA	30	21 (70.0)	62.8 ± 8.5	17 (56.7)	28 (93)	3 (3–5) ^a^	12	30
Alipour et al. (21)	Single-center, prospective	COA	62	40 (64.5)	64 ± 8	23 (37.1)	50 (80.6)	3.0 (2.75–4.00) ^a^	38^c^	196.3
Calvo et al. (22)	Single-center, prospective	COA	35	25 (71.4)	70 ± 7	25 (71.4)	24 (68.6)	3.1 + 1.1	13	37.9
Romanov et al. (23)	Single-center, RCT	COA	45	28 (62.2)	60 ± 5	21 (46.7)	NA	2.2 ± 0.6	24	78
Panikker et al. (24)	Multicenter, prospective	COA	20	13 (65.0)	68 ± 7	20 (100.0)	NA	3.1 ± 1.2	12	20
Phillips et al. (25)	Single-center, prospective	COA	98	67 (68.4)	65 ± 7	42 (42.9)	NA	2.6 ± 1.0	26.7	218.3
Pelissero et al. (26)	Single-center, prospective	COA	21	14 (66.7)	66.9 ± 10.4	17 (81.0)	21 (100.0)	2.8 ± 1.22	14.93	26.1
Wintgens et al. (27)	Multicenter, prospective	COA	349	202 (57.9)	63.1 ± 8.2	152 (43.6)	NA	3.0 (2.0–4.0) ^a^	34.5^c^	1,003.4
Fassini et al. (28)	Single-center, prospective	COA	49	32 (65.3)	69 ± 8	24 (49.0)	16 (22.9)	2.8 ± 1.2	24	98
Liu et al. (29)	Multicenter, prospective	COA	50	33 (66.0)	64.9 ± 7.7	23 (46.0)	30 (60.0)	3.7 ± 1.4	20.2	84.2
Du et al. (17)	Multicenter, retrospective	COA	122	73 (59.8)	66.4 ± 8.8	61 (50.0)	122 (100.0)	4.3 ± 1.4	11.5	116.9
Chen et al. (30)	ingle-center, prospective	COA	178	94 (52.8)	68.9 ± 8.1	90 (50.6)	78 (43.8)	3.3 ± 1.5	12	72
Kita et al. (31)	Single-center, retrospective	COA	42	28 (66.7)	71.1 ± 8.5	NA	NA	3.3 ± 1.1	18.6	65.1
Liu et al. (32)	Single-center, prospective	COA	27	20 (74.1)	64.7 ± 6.3	10 (37.0)	NA	4.8 ± 1.4	18	40.5
Mo et al. (33)	Single-center, retrospective	COA	76	39 (51.3)	69.9 ± 7.9	39 (51.3)	NA	3.6 ± 1.3	24	152
Phillips et al. (34)	Multicenter, prospective	COA	142	77 (54.2)	64.2 ± 7.2	43 (30.3)	NA	3.4 ± 1.4	24.2	286.4
Ren et al. (35)	Single-center, retrospective	COA	76	47 (61.8)	67.0 ± 7.5	25 (32.9)	37 (48.7)	3.4 ± 1.9	23.7	150.1
Chen et al. (36)	Single-center, prospective	COA	56	32 (57.1)	69.4 ± 7.5	0 (0)	NA	4.0 (3.0–5.0) ^a^	12	56
Mokracek et al. (18)	Single-center, retrospective	TCA	30	20 (66.7)	NA	28 (93.3)	NA	1.7 (0–4) ^b^	11.09	20.9
Ellis et al. (37)	Single-center, retrospective	TCA	65	50 (76.9)	64.5 ± 8.8	53 (81.5)	57 (86.7)	2.48 ± 1.54	34.3	183
van Laar et al. (38)	Multicenter, prospective	TCA	222	152 (68.5)	66 ± 9	179 (80.6)	NA	2.3 ± 1.0	20^c^	369
Osmancik et al. (39)	Single-center, prospective	TCA	40	23 (57.5)	62.6 ± 8.6	40 (100.0)	NA	2.2 ± 1.47	12.1	40.3
Salzberg et al. (40)	Single-center, prospective	TCA	42	14 (33.3)	65 ± 7	27 (64.3)	38 (90.5)	2 (0–4) ^b^	20	70
Haldar et al. (41)	Multicenter, RCT	TCA	55	44 (80.0)	63.8 ± 8.9	55 (100.0)	NA	NA	12	55

persAFib, persistent AFib; LSPAFib, longstanding persistent AFib; OAC, oral anticoagulation; NA, not applicable.

^a^Median (with interquartile range) was reported. ^b^Median (with minimum and maximum values) was reported. ^c^Median was reported.

Differences in baseline characteristics between COA and TCA studies were presented in Table 2. Age (66.2 versus 64.7 years, *p* = 0.209), male gender distribution (61.7 versus 64.6%, *p* = 0.486), left ventricular ejection fraction (LVEF; 61.3 versus 58.5%, *p* = 0.209) and comorbidities including hypertension (75.5 versus 67.6%, *p* = 0.162) and diabetes mellitus (19.8 versus 20.4%, *p* = 0.729) were comparable between two groups. The left atrial dimension was significantly larger in TCA group compared to COA group (48.3 versus 43.4 mm, *p* = 0.009). The percentage of persistent AFib and (or) longstanding persistent AFib was significantly lower in COA studies (48.3 versus 87.9%, *p* < 0.001). Furthermore, the CHADS2-VASc score (3.3 versus 2.3, *p* = 0.012) and the percentage of prior stroke/TIA (52.5 versus 57.9%, *p* = 0.005) was higher in patients undergoing COA. The supplementary baseline characteristics were summarized in Supplementary Table 3.

**TABLE 2:** Differences in characteristics between COA and TCA studies at baseline.

Characteristics	COA	TCA	*p*-value
Age (years)	66.2 (64.8–67.6)	64.7 (63.5–65.9)	0.209
Male (%)	61.7 (58.5–64.8)	64.6 (52.9–76.2)	0.486
PersAFib and (or) LSPAFib (%)	48.3 (32.2–64.3)	87.9 (70.0–95.8)	<0.001
Hypertension (%)	75.5 (71.0–80.1)	67.6 (55.6–79.6)	0.162
Diabetes mellitus (%)	19.8 (17.0–22.5)	20.4 (10.5–30.3)	0.729
CHADS2-VASc score	3.3 (2.9–3.6)	2.3 (2.2–2.4)	0.012
LVEF (%)	61.3 (59.7–62.9)	58.5 (52.7–64.3)	0.209
Left atrial dimension (mm)	43.4 (41.7–45.1)	48.3 (43.9–52.8)	0.009
Prior stroke/TIA (%)	39.8 (29.4–50.1)	10.1 (7.2–12.9)	0.005

Data are presented as means or proportions followed by the 95% confidence interval.

P-value of the meta-regression was computed using the metric “Untransformed Proportion” in a binary random-effects model using study type as covariate factor.

PersAFib, persistent atrial fibrillation; LSPAFib, longstanding persistent atrial fibrillation; LVEF, left ventricular ejection fraction; TIA, transient ischemic attack.

The methodological quality of 23 non-randomized controlled studies was assessed by the MINORS tool. Twenty studies with non-comparative design were evaluated using 8 items and 3 comparative studies were assessed with an additional 4 criteria. Based on grading standards, quality was assessed as moderate for 21 studies and 2 studies had a high risk of bias (Supplementary Table 4). Notably, no study had a prospective calculation of study size and an unbiased assessment of endpoints, since most studies were single-arm studies. The remaining 2 study was RCTs and the overall risk of bias evaluated using the RoB 2 tool was “some concern” and “low risk,” respectively (Supplementary Table 5).

### Primary outcome

**FIGURE 2:**
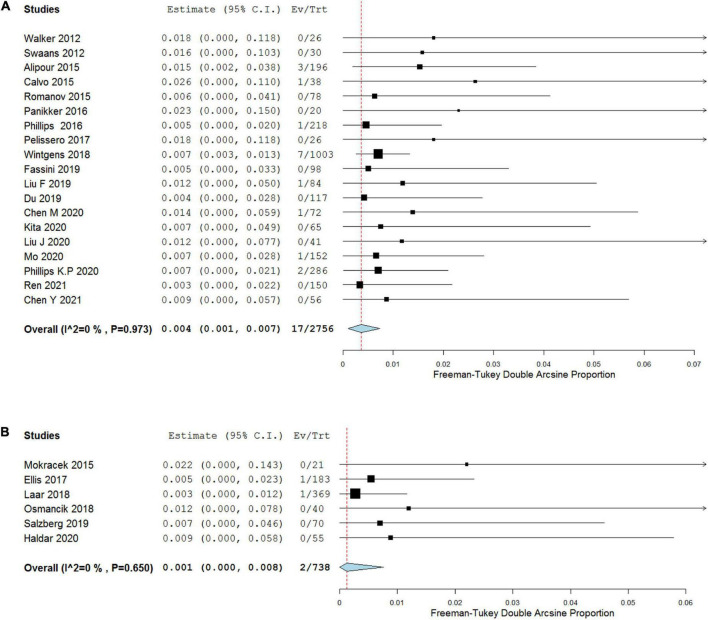
Forest plots depicting primary outcome of COA **(A)** versus TCA **(B)**.

Stroke during the follow-up period of at least 12 months was reported in all included articles. The incidences reported among most included studies were 0, and the highest was 2.6 per 100 patient-years. The pooled incidences of stroke in COA and TCA groups were 0.4 (95% CI, 0.1–0.7) and 0.1 (95% CI, 0–0.8) per 100 patient-years, respectively, and no considerable heterogeneity was found (*I*^2^ = 0%). Meta-regression analysis showed that stroke was comparable between COA and TCA groups (*p* = 0.504; Figure 2 and Table 3).

**TABLE 3:** Differences in outcomes and complications between COA and TCA studies.

	COA		TCA		*p*-value
		
	Number of events (*n*)	95% CI	Number of events(*n*)	95% CI	
**Primary outcome**					
Stroke during follow-up	17	0.4(0.1–0.7) per 100 patient-years	2	0.1 (0–0.8) per 100 patient-years	0.504
**Secondary outcome**					
Acute success rate	1,491	99.1% (98.2–99.8)	216	93.9% (89.2–97.4)	0.001
Postoperative stroke/TIA	2	0.3% (0–0.8)	0	0.3% (0–1.3)	0.948
Postoperative hemorrhage	51	3.0% (2.1–4.0)	7	1.6% (0.2–4.0)	0.023
Postoperative pericardial effusion	26	1.4% (0.7–2.2)	0	0.3% (0.0–1.3)	0.063
All-cause mortality during follow-up	10	0.2 (0–0.6) per 100 patient-years	4	0.3 (0–1.1) per 100 patient-years	0.611

Data were presented as total number of patients of COA or TCA group per event, followed by means and 95% CIs in a binary random-effects model. Statistical analysis for primary outcome and secondary outcomes: one-arm meta-regression “Freeman-Tukey Double Arcsine Proportion” using the study type as a covariate to compare the outcomes between two groups. TIA, transient ischemic attack.

### Secondary outcome

#### Acute success rate

**FIGURE 3:**
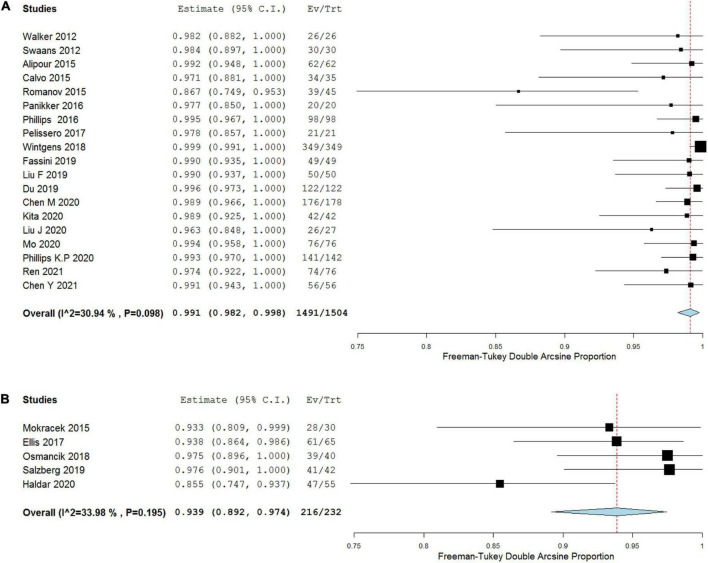
Forest plots depicting acute success rate of COA **(A)** versus TCA **(B)**.

There were 19 COA studies and 5 TCA studies that examined the acute success rate and were therefore suitable for meta-analysis. The success rates of both groups were 99.1% (95% CI, 98.2–99.8%, *I*^2^ = 30.94%, *p* = 0.098) in COA group and 93.9% (95% CI, 89.2–97.4%, *I*^2^ = 33.98%, *p* = 0.195) in TCA group. The pooled results showed COA group had a higher acute success rate compared with TCA group (*p* = 0.001; Figure 3 and Table 3). Two studies (23, 41) showed relatively poor results (86.7 and 85.5%, respectively). The efficiency of complete LAA closure during long-term follow-up could not be calculated because of incomplete reported data in most included studies.

#### All-cause mortality during follow-up period

**FIGURE 4:**
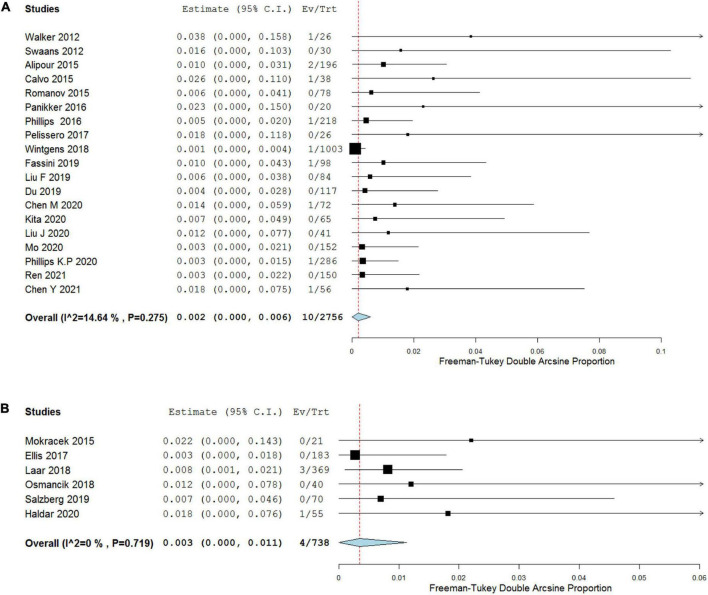
Forest plots showing pooled analysis of all-cause mortality of COA **(A)** versus TCA **(B)** during follow-up period.

All-cause mortality during the follow-up period of at least 12 months was 0.2 per 100 patient-years (95% CI, 0.0–0.6) in COA group as well as 0.3 per 100 patient-years (95% CI, 0.0–1.1) in TCA group and two groups were not statistically different regarding all-cause mortality (*p* = 0.611; Figure 4 and Table 3).

#### Postprocedural complications

A total of 25 studies reported the occurrence of postprocedural stroke/TIA, hemorrhage, and pericardial effusion events. The meta-analysis demonstrated a similar risk of postprocedural stroke/TIA between COA and TCA groups (0.3 versus 0.3%, *p* = 0.948; Supplementary Figure 1 and Table 3). Postprocedural hemorrhage events including all major or minor hemorrhage complications after the procedure were reported with incidences ranging from 0.0 to 10.0%. The pooled results of COA and TCA groups were 3.0% (95% CI, 2.1–4.0%) and 1.6% (95% CI, 0.2–4.0%) respectively, and meta-regression analysis showed a higher risk of hemorrhage of COA group during postprocedural period compared with TCA group (*p* = 0.023; Supplementary Figure 2 and Table 3). By pooling the results of postprocedural pericardial effusion, the incidences of COA and TCA groups were 1.4% (95% CI, 0.7–2.2%) and 0.3% (95% CI, 0–1.3%) respectively, and did not differ statistically between two groups (*p* = 0.063; Supplementary Figure 3 and Table 3). No patients died during the postprocedural period.

## Discussion

Our meta-analysis was the first to compare the safety and efficiency outcomes of COA versus TCA in patients with AFib. No significant difference in stroke prevention was found in patients undergoing COA versus TCA after at least 12-month follow-up. COA and TCA were similar in terms of all-cause mortality during the follow-up period. The COA group had a higher acute success rate than TCA group. COA placed the patients at a higher risk of hemorrhage during the perioperative period. However, postprocedural complications (stroke/TIA and pericardial effusion) and mortality were comparable between the two groups.

Catheter ablation has been shown more efficacious in rhythm controlling of AFib than AADs, with added benefit of reducing medical burden and improving quality of life (42, 43). Recent evidence demonstrated that surgical ablation during intra-cardiac surgery is more effective in rhythm control than catheter ablation. However, surgical ablation is more invasive with higher postoperative complication rates and a longer duration of hospitalization (44, 45). Hybrid ablation, combining thoracoscopic epicardial and transvenous endocardial ablation procedure, has illustrated more favorable outcomes. In a recent meta-analysis of single-arm studies including 3,716 patients (538 undergoing hybrid ablation and 3,178 undergoing catheter ablation), hybrid ablation was more effective than catheter ablation in maintaining sinus rhythm (SR) in patients with persistent or longstanding persistent AFib (10).

Clinical maintenance of SR does not reduce the frequency of thrombotic events. The AFFIRM (46) and RACE (47) trials have demonstrated that the incidence of thrombotic events was not altered in patients undergoing rate versus rhythm control by AADs. Even if SR is maintained with AADs, long-term anticoagulation is required. For isolated LAA closure, a meta-analysis (48) including 3 randomized open-label controlled trials (PROTECT AF, PREVAIL, and PRAGUE-17) showed that LAA closure was non-inferior or superior to vitamin K antagonist for all stroke or systemic embolism. Thus, LAA closure with AF ablation aiming at concomitant stroke prevention as well as rhythm control might be a more comprehensive treatment. In recent years, various studies have reported that both COA and TCA are feasible and safe. A recent cohort study (33) using propensity score matching was performed to compare the procedural and long-term outcomes of COA with isolated CA or isolated left atrial appendage closure (LAAO). This study included patients who underwent COA (combined group), CA alone (catheter ablation-only group), or LAAO alone (LAAO-only group). The AFib-free rate of COA group was comparable with that of CA-only group. Compared with LAAO-only group, COA group achieved similar complete occlusion rates immediately after the procedure and 45 days after the procedure. And incidences of stroke and bleeding events of COA group were lower compared with those of CA-only group and LAAO-only group. Stroke events reported in most studies included in our work were clinically rare, further indicating the combined procedure is safe and efficacious. In addition, surgical LAA clipping might provide an “electrical isolation” effect, thereby potentially reducing the stroke risk (11). However, relevant evidence is currently scarce regarding the comparison between catheter LAA closure and surgical LAA clipping. And future investigation comparing the effectiveness and safety of ablation combined with LAA closure with isolated LAA closure or isolated ablation is necessary.

There was no evidence for device thrombus in any patients who had stroke events in COA group. Most stroke events were thought related to incomplete LAA closure. In addition, almost all stroke events occurred after OAC was discontinued. Except for LAA, apical thrombus after myocardial infarction was found in two patients. Alipour et al. (21). reported that 3 patients had plaque formation in carotid arteries, and 1 patient had > 50% stenosis in bilateral carotid arteries. In TCA group, one stroke event occurred when one patient was receiving OAC and in sinus rhythm (38). Another one occurred with confirmed complete LAA closure, without OAC use and cardiac thrombus (37). All-cause mortality during the follow-up period of two groups was also extremely low and only a small fraction of mortality events were related to the cardiac cause or OAC use.

COA had a higher acute success rate than TCA. In COA group, the main rationale for procedural failure was that the occlusion device did not match the size of the LAA. Whereas in TCA group, the procedural failure of LAA closure was mainly attributed to lung or cardiac adhesions or unique anatomical features. However, as a consequence of incomplete reporting of data, the long-term efficacy of the LAA closure could not be measured.

Our meta-analysis reported most postoperative complications available for comparative analysis. In COA group, patients had a higher risk of hemorrhage during the perioperative period in comparison to TCA group. Postoperative hemorrhage events were mainly minor in COA group, such as groin hematoma. The majority of hemorrhage events occurring in TCA group were considered due to bleeding from the thoracic wall incision, which required transfusion or surgical intervention. Although no mortality happened during the postprocedural period, 25 pericardial effusion events including cardiac tamponade developed in COA group. Whereas in TCA group, no pericardial effusion event was reported. One patient had a tear in the right upper pulmonary vein during encircling with the dissector in TCA group. Finally, as previously mentioned, postoperative stroke/TIA rarely occurred in both groups.

Several limitations in this meta-analysis should be paid attention to interpret the results. Firstly, only 2 RCTs have been found by our digital search and included in this meta-analysis. Secondly, this meta-analysis contained a relatively small sample size. Thirdly, observational studies inevitably introduced a source of bias due to their non-randomized, unblinded design. Fourthly, differences between the included studies in the percentages of long-standing persistent AFib, antithrombotic agents, and other variables have been several possible confounders. Fifthly, the arrhythmia-free survival could not be meta-analyzed due to the incompetent data at the end of follow-up or at a specific time point. Finally, evidence is missing regarding the mid-term and long-term efficacy of COA versus TCA.

In summary, this meta-analysis demonstrated that the safety and efficacy endpoints of COA and TCA in patients with AFib are similar. However, data directly comparing both techniques are lacking. Large randomized clinical trials investigating the optimal strategy for AFib and efficacy and safety outcomes of different procedures are warranted in the near future.

## Data availability statement

The raw data supporting the conclusions of this article will be made available by the authors, without undue reservation.

## Author contributions

CZ and XM: concept and design. SZ, YC, JL, YY, HT, HF, and XM: acquisition, analysis, or interpretation of data. SZ, YC, HZ, and XM: drafting of the manuscript. All authors: critical revision of the manuscript for important intellectual content.

## Abbreviations

AFib: atrial fibrillation
AADs: antiarrhythmic drugs
CA: catheter ablation
SA: surgical ablation
OAC: oral anticoagulation
LAA: left atrial appendage
COA: catheter left atrial appendage occlusion combined with ablation
TCA: thoracoscopic surgical left atrial appendage clipping combined with ablation
MOOSE: Meta-analysis of Observational Studies in Epidemiology guidelines
TIA: transient ischemic attack
MINORS: Methodological Index for Non-Randomized Studies
RCTs: randomized controlled trials
RoB 2: Revised Cochrane risk of bias tool for randomized trials
TEE: transesophageal echocardiography
SR: sinus rhythm
LAAO: left atrial appendage closure.
