# Supplementary material for: Concomitant transcatheter occlusion versus thoracoscopic surgical clipping for left atrial appendage in patients undergoing ablation for atrial fibrillation: A meta-analysis
Source: Front Cardiovasc Med. 2022 Sep 6;9:970847. doi: 10.3389/fcvm.2022.970847 (PMC9485627; doi:10.3389/fcvm.2022.970847)
Supplement: Supplementary file 1 [file Data_Sheet_3.PDF]

A

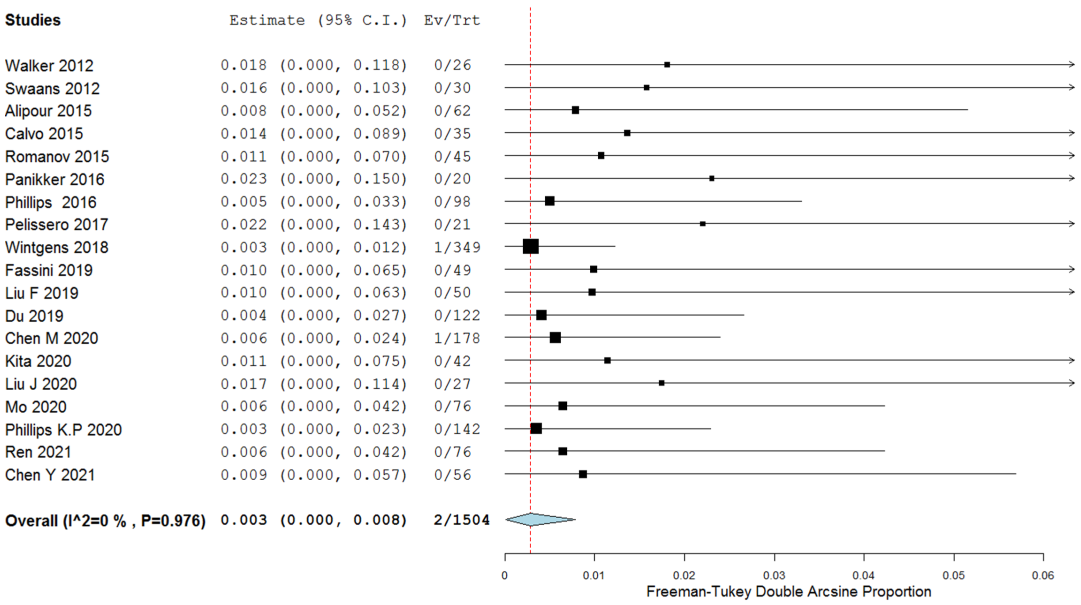

B

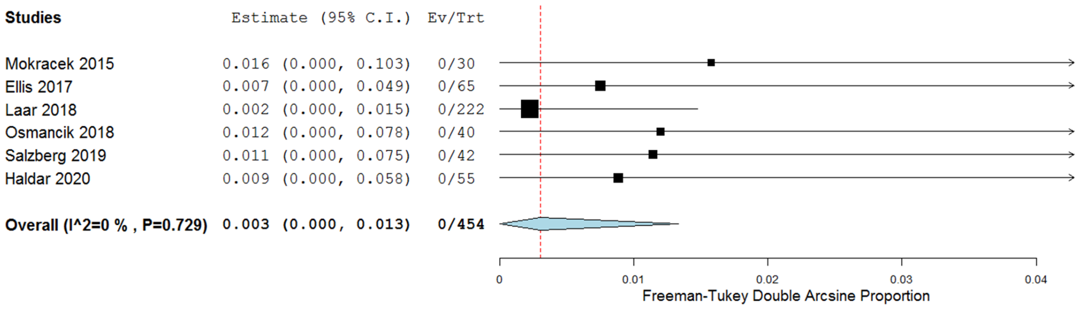

Supplementary Figure 1. Forest plots showing meta-analysis of postoperative stroke/TIA of two techniques. A: COA; B: TCA.

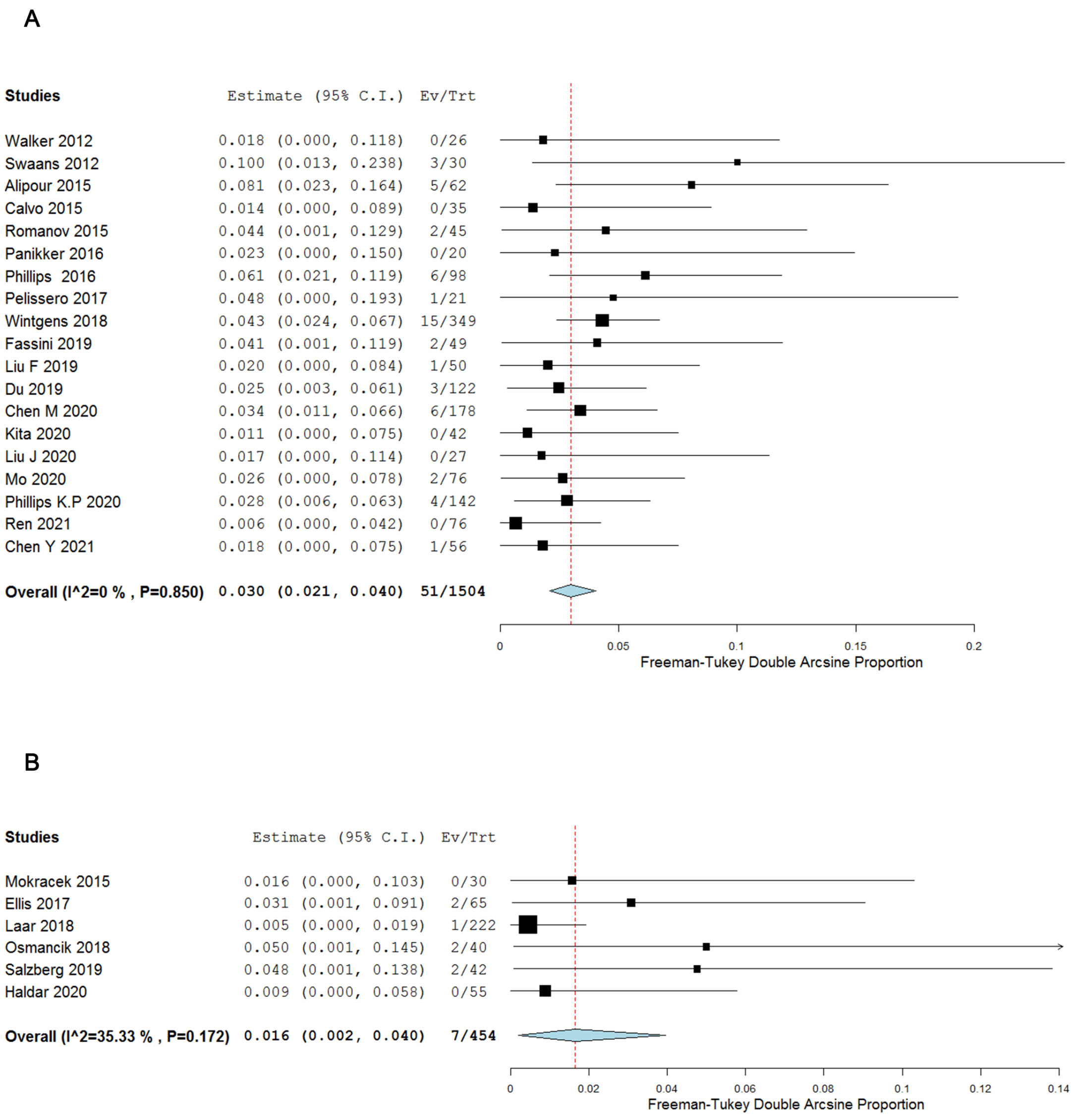

Supplementary Figure 2. Forest plots showing meta-analysis of postoperative hemorrhage of two techniques. A: COA; B: TCA.

A

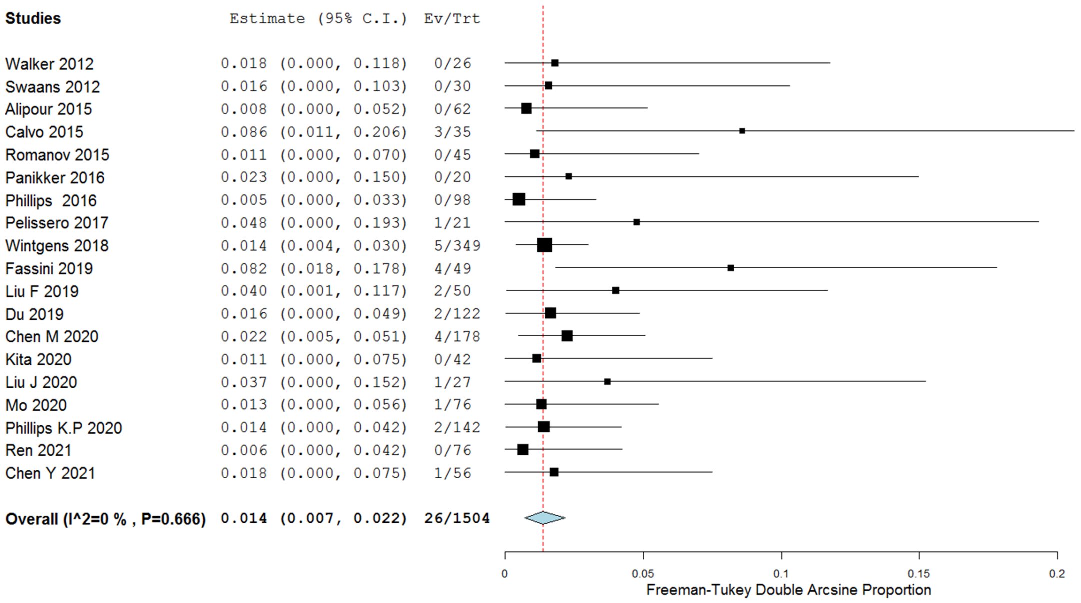

B

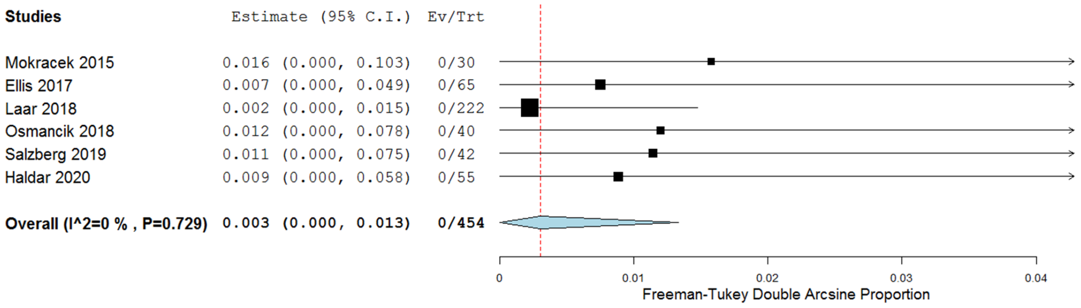

Supplementary Figure 3. Forest plots showing meta-analysis of postoperative pericardial effusion of two techniques. A: COA; B: TCA.
