# Supplementary material for: Concomitant transcatheter occlusion versus thoracoscopic surgical clipping for left atrial appendage in patients undergoing ablation for atrial fibrillation: A meta-analysis
Source: Front Cardiovasc Med. 2022 Sep 6;9:970847. doi: 10.3389/fcvm.2022.970847 (PMC9485627; doi:10.3389/fcvm.2022.970847)
Supplement: Supplementary file 2 [file Data_Sheet_4.docx]

**Supplementary Table 1. MOOSE Checklist**

| **Reporting Criteria** | **Reported (Yes/No)** | **Reported on Page Number** |
| --- | --- | --- |
| Problem definition | Yes | 3 |
| Hypothesis statement | Yes | 3 |
| Description of study outcomes | Yes | 3 |
| Type of exposure or intervention used | Yes | 3 |
| Type of study designs used | Yes | 3 |
| Study population | Yes | 3 |
| Qualifications of searchers (e.g. librarians and investigators) | No | - |
| Search strategy, including time period included in the synthesis and keywords | Yes | 4 |
| Effort to include all available studies, including contact with authors | Yes | 4 |
| Databases and registries searched | Yes | 4 |
| Search software used, name and version, including special features used (e.g. explosion) | Yes | 4 |
| Use of hand searching (e.g. reference lists of obtained articles) | Yes | 4 |
| List of citations located and those excluded, including justification | Yes | 4 |
| Methods of addressing articles published in languages other than English | Yes | 3 |
| Method of handling abstracts and unpublished studies | Yes | 3 |
| Description of any contact with authors | Yes | No contact with authors |
| Description of relevance or appropriateness of studies assembled for assessing the hypothesis to be tested | Yes | 4 |
| Rationale for the selection and coding of data (e.g. sound clinical principles or convenience) | Yes | 4 |
| Documentation of how data were classified and coded (e.g. multiple raters, blinding, and interrater reliability) | Yes | 4 |
| Assessment of confounding (e.g. comparability of cases and controls in studies where appropriate) | No | - |
| Assessment of study quality, including blinding of quality assessors; stratification or regression on possible predictors of study results | Yes | 4,5 |
| Assessment of heterogeneity | Yes | 5 |
| Description of statistical methods (e.g. complete description of fixed or random effects models, justification of whether the chosen models account for predictors of study results, dose-response models, or cumulative meta-analysis) in sufficient detail to be replicated | Yes | 4,5 |
| Provision of appropriate tables and graphics | Yes | Throughout manuscript |
| Graphic summarizing individual study estimates and overall estimate | Yes | Figures 2–4, Figure S1-S3 |
| Table giving descriptive information for each study included | Yes | Table 1, Table S2-S3 |
| Results of sensitivity testing (e.g. subgroup analysis) | No | - |
| Indication of statistical uncertainty of findings | Yes | 6,7 |
| Quantitative assessment of bias (e.g. publication bias) | No | - |
| Justification for exclusion (e.g. exclusion of non-English-language citations) | Yes | 3-5, Figure 1 |
| Assessment of quality of included studies | Yes | 5,6, Table S4-S5 |
| Consideration of alternative explanations for observed results | Yes | 7,8 |
| Generalization of the conclusions (i.e. appropriate for the data presented and within the domain of the literature review) | Yes | 7,8 |
| Guidelines for future research | Yes | 7,8 |
| Disclosure of funding source | Yes | 9 |

**Supplementary Table 2. Ablation and LAA closure strategy**

| **Source** | **LAA closure device** | **Lesions** | **Ablation approach** |
| --- | --- | --- | --- |
| Walker 2012 | Watchman | PVI±CFAE or lines | Radiofrequency |
| Swaans 2012 | Watchman | PVI±CFAE | Radiofrequency |
| Alipour 2015 | Watchman | PVI±CFAE | Radiofrequency |
| Calvo 2015 | Watchman or ACP | PVI±lines | Radiofrequency |
| Romanov 2015 | Watchman | PVI | Radiofrequency |
| Panikker 2016 | Watchman | PVI+ lines+ LAAEI | Radiofrequency |
| Phillips 2016 | Watchman | PVI±CFAE or lines | Radiofrequency |
| Pelissero 2017 | Watchman or ACP | PVI | Radiofrequency or Cryoballoon |
| Wintgens 2018 | Watchman | PVI±CFAE or lines or SVC or GP | Radiofrequency |
| Fassini 2019 | Watchman or ACP | PVI | Cryoballoon |
| Liu F 2019 | Watchman or ACP | PVI | Radiofrequency or Cryoballoon |
| Du 2019 | Watchman or ACP | PVI±lines | Radiofrequency |
| Chen M 2020 | Watchman | PVI±lines | Radiofrequency |
| Kita 2020 | Watchman | PVI+LAAEI or lines | Radiofrequency |
| Liu J 2020 | Watchman or ACP or LAmbre | PVI | Cryoballoon |
| Mo 2020 | Watchman | PVI±CFAE or lines | Radiofrequency |
| Phillips K.P 2020 | Watchman | PVI±lines | Radiofrequency |
| Ren 2021 | Lefort or Lacbes or Watchman | PVI | Cryoballoon |
| Chen Y 2021 | LAmbre | PVI | Radiofrequency |
| Mokracek 2015 | AtriClip | Box lesion+lines | Hybrid |
| Ellis 2017 | AtriClip | PVI+lines | Hybrid |
| Laar 2018 | AtriClip | Box lesion | Hybrid or Epicardial |
| Osmancik 2018 | AtriClip | Box lesion | Epicardial |
| Salzberg 2019 | AtriClip | PVI | Epicardial |
| Haldar 2020 | AtriClip | Box lesion+lines | Epicardial |

LAA = left atrial appendage, ACP = Amplatzer Cardiac Plug, Box lesion = pulmonary vein isolation, roof line and inferior line. PVI = pulmonary vein isolation, CFAE = complex fractionated atrial electrograms, LAAEI = left atrial appendage electrical isolation, GP = ganglionated plexis, SVC = superior vena cava.

**Supplementary Table 3 Baseline characteristics**

| **Source** | **Left atrial dimension (mm)** | **LVEF (%)** | **HAS-BLED Score** | **Hypertension (%)** | **Diabetes mellitus (%)** | **Prior stroke/TIA (%)** |
| --- | --- | --- | --- | --- | --- | --- |
| Walker 2012 | - | 62 ± 5 | - | 20 (77) | 5 (19) | 11 (42) |
| Swaans 2012 | - | - | 2 (1–3) | - | - | 9 (30) |
| Alipour 2015 | - | - | 2.0 (2.0–3.0) | - | - | 48 (77.4) |
| Calvo 2015 | - | - | 3.1+1 | 26 (74) | - | 3 (9) |
| Romanov 2015 | 49 ± 6 | 62 ± 5 | 3.5 ± 0.8 | 38 (84) | 9 (20) | 4 (9) |
| Panikker 2016 | 46 ± 3 | - | 2.5 ± 1.1 | - | - | - |
| Phillips 2016 | - | 61 ± 8 | 1.9 ± 0.8 | 64(65) | 16(16) | 28(29) |
| Pelissero 2017 | - | - | 3.2 ± 0.8 | 14(66.7) |  | 2(9.5) |
| Wintgens 2018 | - | 60.3 ± 8.2 | 3.0 (2.0–3.0) | 227 (65.0) | 57 (16.3) | 170 (48.7) |
| Fassini 2019 | - | 54 ± 5 | 3 ± 1 | 39 (80) | 11 (23) | 23 (47) |
| Liu F 2019 | 38.6 ± 5.1 | 63.9 ± 9.0 | 2.5 ± 0.9 | 42 (84) | 11 (22) | 33 (66) |
| Du 2019 | 42.1 ± 6.1 | 62.0 ± 7.1 | 3.3 ± 1.0 | 85 (69.7) | 26 (21.3) | 82 (67.2) |
| Chen M 2020 | 42.5 ± 5.6 | 64.0 ± 5.9 | 1.6 ± 1.0 | 129 (72.5) | 39 (21.9) | 50 (28.1) |
| Kita 2020 | - | 57.5 ± 9.9 | 2.5 ± 1.4 | 37 (88.0) | 10 (24.4) | 4 (9.8) |
| Liu J 2020 | 40.3± 5.2 | 61.2± 3.9 | 3.6 ± 1.3 | 25(92.6) | 12(44.4) | 23(85.2) |
| Mo 2020 | 42.7 ± 5.7 | 63.9 ± 6.3 | 3.3 ± 1.1 | 56 (73.7) | 14 (18.4) | 23 (30.3) |
| Phillips K.P 2020 | - | - | 1.5 ± 0.9 | 114 (80.3) | 19 (13.4) | 57 (40.1) |
| Ren 2021 | 44.9 ± 5.5 | 60.2 ± 7.4 | 2.3 ± 1.1 | 49 (64.5) | 17 (22.4) | 34 (44.7) |
| Chen Y 2021 | 44.7 ± 4.8 | 64.8 ± 10.1 | 3.0 (2.0–3.0) | 39 (69.6) | 16 (28.6) | 26 (46.4) |
| Mokracek 2015 | 48 ± 5 | 64 ± 8 | - | - | - | - |
| Ellis 2017 | 56.0 ± 7.5 | - | 2.22 ± 1.24 | 51(78.5) | 15(23.1) | 7 |
| Laar 2018 | - | - | - | - | - | 22 (9.9) |
| Osmancik 2018 | 44.9 ± 8.8 | 52.3 ± 12.3 | - | 21 (52.5) | 9 (22.5) | 8 (20) |
| Salzberg 2019 | 48 ± 10 | 60 (40-77) | - | 32 (76) | 13 (31) | 4 (10) |
| Haldar 2020 | 44.7 ± 5.8 | 58.8 (8.7) | - | 33 (55) | 5 (8.3) | 4 (6.7) |

LVEF: left ventricular ejection fraction; TIA: transient ischemic attack.

**Supplementary Table 4. Assessment of the quality of non-randomized controlled trials studies according to MINORS tool.**

| Authors | Q1 | Q2 | Q3 | Q4 | Q5 | Q6 | Q7 | Q8 | Q9 | Q10 | Q11 | Q12 | Score (quality) |
| --- | --- | --- | --- | --- | --- | --- | --- | --- | --- | --- | --- | --- | --- |
| Walker 2012 | 2 | 2 | 2 | 2 | 0 | 2 | 1 | 0 |  |  |  |  | 11 (Intermediate) |
| Swaans 2012 | 2 | 2 | 2 | 2 | 0 | 2 | 2 | 0 |  |  |  |  | 12 (Intermediate) |
| Alipour 2015 | 2 | 2 | 2 | 2 | 0 | 2 | 2 | 0 |  |  |  |  | 12 (Intermediate) |
| Calvo 2015 | 2 | 2 | 2 | 2 | 0 | 2 | 2 | 0 |  |  |  |  | 12 (Intermediate) |
| Panikker 2016 | 2 | 2 | 2 | 2 | 0 | 2 | 2 | 0 | 0 | 2 | 2 | 2 | 18 (Intermediate) |
| Phillips 2016 | 2 | 2 | 2 | 2 | 0 | 2 | 2 | 0 |  |  |  |  | 12 (Intermediate) |
| Pelissero 2017 | 2 | 1 | 2 | 2 | 0 | 2 | 0 | 0 | 0 | 2 | 2 | 2 | 15 (Intermediate) |
| Wintgens 2018 | 2 | 2 | 2 | 2 | 0 | 2 | 0 | 0 |  |  |  |  | 10 (Intermediate) |
| Fassini 2019 | 2 | 1 | 2 | 2 | 0 | 2 | 2 | 0 |  |  |  |  | 11 (Intermediate) |
| Liu F 2019 | 2 | 2 | 2 | 2 | 0 | 2 | 2 | 0 |  |  |  |  | 12 (Intermediate) |
| Du 2019 | 2 | 2 | 0 | 2 | 0 | 2 | 0 | 0 |  |  |  |  | 8 (low) |
| Chen M 2020 | 2 | 2 | 2 | 2 | 0 | 2 | 0 | 0 |  |  |  |  | 10 (Intermediate) |
| Kita 2020 | 2 | 0 | 2 | 2 | 0 | 2 | 2 | 0 |  |  |  |  | 10 (Intermediate) |
| Liu J 2020 | 2 | 2 | 2 | 2 | 0 | 2 | 1 | 0 |  |  |  |  | 11 (Intermediate) |
| Mo 2020 | 2 | 2 | 0 | 2 | 0 | 2 | 1 | 0 | 0 | 2 | 2 | 2 | 15 (Intermediate) |
| Phillips K P 2020 | 2 | 2 | 2 | 2 | 0 | 2 | 2 | 0 |  |  |  |  | 12 (Intermediate) |
| Ren 2021 | 2 | 2 | 0 | 2 | 0 | 2 | 2 | 0 |  |  |  |  | 10 (Intermediate) |
| Chen Y 2021 | 2 | 2 | 2 | 2 | 0 | 2 | 2 | 0 |  |  |  |  | 12 (Intermediate) |
| Mokracek 2015 | 1 | 1 | 0 | 1 | 0 | 2 | 2 | 0 |  |  |  |  | 7 (low) |
| Ellis 2017 | 2 | 2 | 2 | 2 | 0 | 2 | 2 | 0 |  |  |  |  | 12 (Intermediate) |
| Laar 2018 | 2 | 2 | 2 | 2 | 0 | 2 | 2 | 0 |  |  |  |  | 12 (Intermediate) |
| Osmancik 2018 | 2 | 2 | 2 | 2 | 0 | 2 | 0 | 0 |  |  |  |  | 10 (Intermediate) |
| Salzberg 2019 | 1 | 2 | 1 | 1 | 0 | 2 | 2 | 0 |  |  |  |  | 9 (Intermediate) |

0 = not reported; 1 = reported but inadequate; 2= reported and adequate.

For non-comparative studies: > 12 = high; 8–12 = intermediate; < 8 = low.

For comparative studies: > 18 = high; 13–18 = intermediate; < 13 = low.

Q1: Did the study have a clearly stated aim?

Q2: Were consecutive patients included?

Q3: Were data collected prospectively?

Q4: Were endpoints appropriate to the study?

Q5: Was there an unbiased assessment of endpoints?

Q6: Was the follow-up period adequate?

Q7: Was loss to follow-up < 5%?

Q8: Was there a prospective calculation of study size?

Q9: Was there an adequate control group?

Q10: Was the control group contemporary?

Q11: Were the baseline characteristics comparable?

Q12: Were statistical analyses adequate?

**Supplementary Table 5. Assessment of the quality of randomized controlled trials according to Cochrane Risk of Bias Tool for Randomized Controlled Trials.**

| **Authors** | **^1^Randomization process** | **^2^Identification and recruitment** | **^3^Intervention deviation** | **^4^Missing outcome data** | **^5^Outcome measurement** | **^6^ Reported results selection** | **Score (quality)** |
| --- | --- | --- | --- | --- | --- | --- | --- |
| Romanov 2015 | Low risk | Some concern | Some concern | Low risk | Low risk | Some concern | Some concern |
| Haldar 2020 | Low risk | Low risk | Low risk | Low risk | Low risk | Low risk | Low risk |
